# Special Fracture Number

**Published:** 1913-12

**Authors:** 


SPECIAL FRACTURE NUMBER.—The *American Journal of Surgery* will present in January an issue of their journal devoted exclusively to Fractures and their treatment. The following subjects will be presented by acknowledged authorities in this special branch of surgical work:

"Astragalus Injuries" by F. J. Cotton, M. D., Boston, Mass. "Diagnosis of Fracture" by Lewis A. Stimson, M. D., New York. "Position in the Treatment of Juxta Epiphyseal Fractures at the Hip and Shoulders" by Fred. Albee, M. D., New York. "A Splint for Maintaining Nail Extension During Transport" by John C. A. Gerster, M. D., New York. "Fracture of the Skull; Roentgen Ray as an Aid in its Diagnosis" by W. H. Luckett, M. D., New York. "Vicious Union" by James K. Young, M. D., Philadelphia, Pa. "The Immediate and Remote Results of Fractures of the Skull and Spine" by Chas. Elsberg, M. D., New York. "Conservation in the Treatment of Fractures" by Wm. L. Estes, M. D., So. Bethlehem, Pa. "Some Phases of Fracture Treatment as Based on Hospital Experience" by E. S. Van Duyn, M. D., Syracuse, N. Y. "The Treatment of Fractures" by E. P. Magruder, M. D., Washington, D. C.

